# 1112. Case Study. Effectiveness of Collagen-elastin Matrix in Reconstructive Burn Surgery: A Case Study

**DOI:** 10.1093/jbcr/irag033.216

**Published:** 2026-04-06

**Authors:** Tait Olaveson

**Affiliations:** The Burn Center at Eastern Idaho Regional Medical Center, Rigby, ID

## Abstract

**Patient Presentation (age range, injury details, relevant history):**

A 33-year-old male sustained 31% total body surface area (TBSA) burns during a grain silo explosion.

**Clinical Challenges:**

His clinical course was complicated by wound infections with environmental organisms uncommon in routine burn care. Rehabilitation was further limited by non-compliance with outpatient therapy, splinting, and follow-up. He subsequently developed a severe recurrent antecubital fossa contracture of the right upper extremity that restricted elbow extension and functional independence.

**Management Approach:**

The patient underwent admission for definitive management. After wide excision of hypertrophic scar tissue, a 1 mm-CEM as well as split-thickness skin graft (STSG, 0.008 inches) were applied over the defect and secured using negative pressure wound therapy (NPWT) at -75 mmHg. NPWT was continued for an additional 11 days. Early physiotherapy was re-initiated on postoperative day (POD) 9.

**Outcomes:**

Preoperatively, elbow extension was limited to 55°, measured by outpatient facility utilizing goniometry. Full extension to 0° was achieved intraoperatively and preserved through POD 9. At graft takedown, 100% graft adherence was noted, without loss or complications. The patient was discharged on POD 21 with instructions for strict adherence to therapy. At 2 months, elbow extension measured 0° and has remained stable at follow-up to date.

**Lessons Learned:**

This case highlights the effectiveness of CEM in reconstructive burn surgery, allowing early grafting, durable functional correction, and shortened hospitalization. With improved adherence to rehabilitation, the patient regained meaningful upper-extremity function after three years of disability.

**Applicability to Practice:**

The use of CEM may change reconstructive burn protocols by accelerating time to graft readiness, reducing morbidity, and improving functional outcomes in high-risk patients, including those with infection, recurrence, or compliance challenges.

